# Advances in the immunosuppression of porcine reproductive and respiratory syndrome virus

**DOI:** 10.3389/fvets.2026.1775261

**Published:** 2026-02-27

**Authors:** Huawei Li, Chen Lv, Ruining Wang, Xuyong Zhao, Liangzong Huang, Mengmeng Zhao

**Affiliations:** 1 Institute of Animal Product Quality and Safety Technology, Henan University of Animal Husbandry and Economy, Zhengzhou, China; 2 Guangdong Provincial Key Laboratory of Animal Molecular Design and Precise Breeding, School of Animal Science and Technology, Foshan University, Foshan, Guangdong, China

**Keywords:** immunosuppression, porcine reproductive and respiratory syndrome, PRRSV, research progress, virus

## Abstract

Porcine reproductive and respiratory syndrome (PRRS) is a highly infectious disease caused by the PRRS virus (PRRSV), its impact is second only to that of African swine fever (ASFV). Since the discovery of this disease, comprehensive studies have been conducted on its genome structure, protein function, pathogenicity, transmission route, and epidemiology as well as vaccines, prevention, and control. Despite the availability of numerous vaccines, complete immune protection in pigs is lacking. This limitation may be attributed to immune evasion, immunosuppression, or inherent characteristics of pigs. From the view of immunosuppression, the antigen escape, delayed neutralization antibody production, T cell immunity, antibody dependence enhancement, dendritic cell function inhibition, regulatory T cell induction and thymic destruction of PRRSV were discussed in this review to better understand PRRSV pathogenesis and inform vaccine development.

## 1 Introduction

Porcine reproductive and respiratory syndrome (PRRS) is a widespread and severe disease that affects pigs and is caused by a virus known as PRRS virus (PRRSV), commonly referred to as “blue-ear disease” (1). This virus can infect swine at any developmental stage. With the ability for continuous recombination and high variability, PRRSV poses a substantial threat to the worldwide pig industry because of its relatively high incidence and fatality rate, resulting in considerable damage (2). During pregnancy, sows can experience serious reproductive complications as a result of infection, including early abortions, temporary symptoms preceding miscarriage, stillbirths, mummified fetuses and malformed fetuses in the later stages of gestation. The main clinical indications in male pigs affected by infection are a decline in sexual desire, a decrease in the quality of semen. The mortality rate among newborn piglets is considerably elevated, presented through an elevated body temperature and notable respiratory symptoms that become apparent shortly after birth. Unfortunately, this frequently leads to mortality within a span of just 1–2 days (3, 4). Piglets suffer from diseases during the perinatal weaning period, with specific clinical manifestations including hyperthermia, rough and unkempt hair coat, anorexia, hematochezia, tachypnea, bluish discoloration of the ears and tail, progressive weight loss, and acute mortality. The first reported case of PRRS dates back to 1987 in the United States. Soon after, outbreaks were documented in Canada in 1988, Germany in 1990, and Poland in 1991 (5). A few years later, it evolved into a pandemic of global proportions. In 1995, PRRS was first discovered in China. Bao-Qing et al. (6), Han-Chun et al. (7), and Ping et al. (8) isolated the virus in subsequent years (1996 and 1997). A publication in 2021 by the International Committee for the Classification of Viruses categorized PRRSV into two primary strains: European and North American. Genetic sequence homology between the two strains is low, ranging from 60 to 70% (9). The European strain was distinctly categorized as PRRSV-1, also known as PRRSV Type 1. The PRRSV variant found in the United States is designated as PRRSV-2 (PRRSV Type 2).

It is essential for the diagnosis, prevention, and treatment of PRRS to understand the antigenicity, pathogenicity of PRRSV strains. There are differences in antigenicity of different strains of PRRSV and can cause immunosuppression, this adversely affects prevention and control for PRRS, as well as research and development endeavors aimed at producing effective vaccines. In addition, the ability of infected pigs to combat the disease is reduced. As a result, the presence of this disease significantly raises the incidence rate of various pathogens like the African swine fever virus (ASFV), porcine circovirus (PCV), and porcine pseudorabies virus (PR) (10, 11). The present review explored the immunosuppressive effects of PRRSV considering antigen evasion, protein–protein interactions, delay in T cell immune response, boost in antibody dependence, impairment of dendritic cells (DCs) function, and promotion of regulatory T cell (Treg) differentiation. Taking into consideration the biology of PRRSV, the review also delves into the delayed production of neutralizing antibodies and the hindrance of natural and adaptive immune responses. This study provides references for the prevention and control of PRRSV infection and vaccine development.

## 2 Biological characteristics of PRRSV

Porcine reproductive and respiratory syndrome virus, a positive-sense RNA-enveloped virus, belongs to the family *Arteriviridae* and genus *BetaArterivirus* (12). The PRRSV genome is approximately 15 kb in size and comprises 10 open reading frames (ORFs), a 5′ untranslated region (5′ UTR), 3′ UTR, and polyadenylation (poly(A)) tail (13). Open reading frames include ORF1a, ORF1b, ORF2a, ORF2b, ORF3, ORF4, ORF5, ORF5a, ORF6, and ORF7. ORF1a and ORF1b constitute approximately 75% of the genome and are primarily responsible for encoding the 13 viral non-structural proteins present, including Nsp1α, Nsp1β, Nsp2 ~ Nsp12. In recent years, studies have shown that most non-structural proteins can inhibit host immune response through different ways, such as inhibiting the secretion of interferon (IFN), inhibiting dendritic cells presenting antigens and so on, resulting in immunosuppression (14, 15). ORF2a, ORF2b, ORF3, ORF4, ORF5, ORF6, and ORF7 encode eight proteins that comprise this structure. These include the N protein found in the nucleocapsid layer and the basic proteins (M proteins), membrane proteins (E proteins), and five glycoproteins (GP2a, GP2b, GP3, GP4, and GP5) in the viral envelope coat proteins. ORF2a is responsible for GP2a synthesis, ORF2b encodes protein E, ORF3 encodes GP3, and ORF4 encodes GP4. ORF5a, positioned between ORF4 and ORF5, and encodes GP5a. ORF5, ORF6, and ORF7 encode the GP5, M, and N proteins, respectively (16). GP2a and GP4 are essential for facilitating communication between glycoproteins and interact with host cytokines, enabling PRRSV to invade vulnerable cells, as the protein responsible for adhesion, it may interact with the PRRSV surface receptor pCD163 on the membranes of alveolar macrophages (17). The interaction between GP5 and M proteins via a disulfide bond enables PRRSV to effectively enter susceptible cells, specifically porcine alveolar macrophages, by binding to the pCD169 receptor on their surface. Moreover, GP2, GP3, and GP4 form heterotrimers that play crucial roles in the duplication process (18–20). During the process of viral replication and insertion into host cells, PRRSV N protein binds to its own genomic RNA and self-interacts to create the core capsid (21, 22). The diverse structural and non-structural PRRSV proteins, in addition to serving multiple functions, contribute to innate immunity of the host and play a critical role in the adhesion process of PRRSV. The accumulation and release of PRRSV is facilitated by the interaction of this virus with other proteins, promoting its invasion of susceptible host cells and enabling it to evade host immune responses (23, 24).

## 3 Ability of PRRSV to suppress the immune system

Currently, PRRSV is mainly transmitted through direct contact and respiratory transmission; following successful infiltration, viral incursion causes harm to the host by damaging the nasal mucosal epithelium, tonsils, and alveolar macrophages of the respiratory system, which results in a decrease in the immune system function of pigs and induces immunosuppressive effects. The majority of pigs encountering their primary PRRSV infection demonstrate acute or subacute symptoms, whereas a minority remain asymptomatic (4). If infected pigs survive the initial one-month period of acute infection, they usually progress toward a persistent infection. Persistent infection is a key distinguishing feature of PRRSV from other viruses. At this point, the presence of the virus in the bloodstream decreases, causing a decrease in the amount of virus present in the pigs that are infected. Viral replication mainly occurs within specific tissue locations and other parts of the immune system, including the tonsils and lymph nodes. Nevertheless, the excretion process of this virus persists for an extended duration (25, 26). Congenital porcine viremia lasts for approximately 210 days, whereas acquired porcine viremia typically lasts for approximately 150 days (27). The time required for commercial pigs to reach their full growth cycle is much shorter than the time required for the initial PRRSV infection and subsequent excretion to end naturally. Currently, there are no conclusive findings explaining why pigs are unable to efficiently eliminate PRRSV. Although other factors are present, immunosuppression and the reduced autoimmune ability of PRRSV are likely the main reasons for the lack of timely and effective immune protection.

### 3.1 Porcine alveolar macrophages (PAMs) facilitate the spread of PRRSV

Porcine alveolar macrophages are the primary target of PRRSV. The virus initially attaches to the surface of macrophages in the nasal mucosa or upper respiratory tract and then enters the PAMs through a process called receptor-mediated endocytosis, causing a cytopathic effect. Subsequently, it disseminates via the bloodstream to various organs and replicates within the mononuclear macrophage system. As PRRSV infection progresses to its advanced stages, the endogenous, extrinsic, and reactive oxygen species pathways can trigger apoptosis and subsequent necrosis in target cells. The effects of PAMs on nonspecific phagocytosis, lysis, processing, and antigen presentation of PRRSV and other pathogens are also affected. The involvement of pCD169 and pCD163 receptors located on the PAMs membrane surface is interconnected with the entry and uncoating processes of PRRSV (28), masking the presence of the PRRSV infection, preventing the appearance of viral proteins on the surface of plasma cells, and consequently impeding the acquisition of virus information by the host immune system and inhibiting the ability of antibody-dependent or complement-mediated cleavage to destroy infected cells (29). Wei et al. (30) discovered that PRRSV infection in PAMs resulted in elevated mRNA levels of interferon-λ1 (IFN-λ1) and interferon-λ3 (IFN-λ3), indicating increased expression of these interferons, with IFN-λ3 showing a stronger upregulation than IFN-λ1. The expression of both proteins was reduced, resulting in a weakened immune response. Liu et al. (31) found that PRRSV suppresses the production of interferon regulatory factor 7 (IRF7) in alveolar macrophages, an outcome related to NSP7. As a versatile transcription factor, IRF7 plays a vital role in the virus-triggered IFN signaling pathway. The activity of IRF7 is regulated by post-translational modifications, include phosphorylation and ubiquitination (32). Natural killer cells (NK cells) can effectively kill virus-infected cells and help the body clear the virus. But Cao et al. (33) found that the ability of natural killer cells to kill PAMs infected with PRRSV was impaired within a timeframe of 6–12 h. Similarly, Renukaradhya et al. (34) observed a considerable decline in the cytotoxic activity of natural killer cells in pigs infected with PRRSV.

### 3.2 Delay in the production of PRRSV-neutralizing antibodies

The activation of a range of signaling pathways is initiated when phagocytes and antigen-presenting cells recognize pathogen-associated molecular patterns through pattern recognition receptors on their surfaces, thus playing a crucial role in the primary mediation of the innate immune system (35). The activation of type I IFNs and factors associated with inflammation is triggered, resulting in a general immune response against foreign microorganisms. Germline-encoded receptors mediate recognition of innate immunity, whereas genetic factors determine receptor specificity. The PRRSV-neutralizing antibodies play a vital role in eliminating the virus from the body and defending against reinfection. However, clinical observations have showed that pigs exhibit a delayed immune response, whether infected with a naturally occurring or intentionally introduced virus. Studies have shown that neutralizing antibodies against the structural protein GP5 appeared late, and the first antibodies against other proteins could not effectively neutralize the virus, resulting in pigs unable to effectively clear the virus in the early stage of infection (36). The involvement of N-glycosylation in the proper formation, localization, and functionality of PRRSV GP5 is crucial (37, 38). Yang et al. (39) suggested that the main epitope responsible for neutralizing PRRSV is located on GP5, whereas GP4 and M proteins also have at least one neutralizing epitope. A linear neutralizing epitope, known as the B epitope, was identified on GP5 of PRRSV-1 using a phage display technique and overlapping peptide synthesis. A linear, non-neutralizing epitope, known as epitope A, has also been identified in the outer functional region of GP5. Epitope A was more immunodominant than epitope B. After viral invasion, the body first produces non-neutralizing antibodies against epitope A, followed by the production of neutralizing antibodies against epitope B. Therefore, it was speculated that the presence of epitope A diminishes the immune response to epitope B, thereby delaying the production of neutralizing antibodies. Epitope A of GP5 may act as an attractant, thereby reducing the immune response to epitope B (40). Additionally, glycosylation sites on and around epitope B may decrease its immunogenicity. Mice immunized with PRRS virions were unable to produce virus-specific neutralizing antibodies, suggesting that PRRS virions exhibit low immunogenicity in heteroanimals. Therefore, it was speculated that the primary neutralization determinant of the virus is located in the N-terminal outer functional region of GP5. Some characteristics of this protein, such as the “decoy” epitope and heteroglycation, conceal the key neutralization sites, thereby hindering or reducing the humoral immune response to the middle region and epitopes of the viral GP5 protein. Neutralization of the N-terminal occlusion is considered one of the primary mechanisms for viral immune evasion and the establishment of persistent infection (41–45).

### 3.3 Inhibition of innate immunity by PRRSV

Upon encountering PRRSV infection, the body promptly activates its innate immune system as a primary defense mechanism. Activation of the innate immune system in the body is crucial for establishing a defense against PRRSV as it effectively impedes the replication and transmission of the virus within the body, thereby supporting the development of adaptive immunity (46). Nevertheless, the inherent defense of the body against PRRSV is remarkably feeble, resulting in delayed initiation of immune responses involving both antibodies and cells. These include toll-like receptors (TLRs), NOD-like receptors, RIG-I-like receptors, AIM2-like receptors, C-type lectin receptors, and cyclic GMP-AMP synthetase (46–48).

The TLRs are a group of proteins that can be used to identify different molecular patterns associated with pathogens (PAMPs), the key signaling domain specific to the TLR system is the toll/interleukin (IL)-1 receptor (TIR) domain, located in the cytoplasmic face and adaptor of each TLR (49). Porcine TLR3, TLR7, and TLR8 play important roles in the PRRSV response (50), they are key components in the recognition and reaction to viral infections. Porcine TLR3 exhibits elevated expression levels in renal tissues, duodenum, and spleen. The expression of TLR7 is moderate in several pig tissues, including in the lungs, spleen, bone marrow, intestines, mesenteric lymph nodes, spleen, liver, and lungs (51). Toll-like receptors and cytoplasmic virus recognition receptors can detect RNA viruses (52, 53). For example, TLR3 can identify double-stranded RNA or RNA produced during the replication of various viruses, and TLR7 (or TLR8 in humans) can recognize single-stranded RNA (54). The TIR domain of TLR3 recruits the adaptor protein TRIF to initiate signaling cascades that trigger the activation of transcription factors, including nuclear factor-kappa B (NF-κB), interferon regulatory factor 3 (IRF3), and activator protein-1, ultimately leading to the induction of IFN-β (55). The N-terminal domain of TRIF binds NF-κB-activated kinase-associated protein 1 and tumor necrosis factor receptor-associated factor 3 to cup-binding kinase-1 I-κB kinase-ε, leading to phosphorylation of IRF3. It then forms a dimer and is transported to the nucleus to induce the expression of IFN-β and other antiviral genes. Unlike TLR3, which uses TRIF as the primary adapter, MyD88 induces the expression of IFN in TLR7/8/9 (56). Signaling begins with the binding of MyD88 to TRAF6 and IRAK-1, leading to the activation of IRF-5 and IRF-7 (57). TLR9 facilitates the interaction between MyD88 and IRF7 through a kinase complex containing TRAF6 and either IRAK-1 or IRAK-4. The IRAK family members play different roles in various cell types (58). The complex of IRF7 and IRAK-1 results in the phosphorylation of IRF7 by IRAK-1. The phosphorylated IRF7 then forms dimers and translocates into the nucleus, primarily to express IFN-α. IKKa is recognized for its role in the activation of NF-κB; therefore, the activation of IRF7 may be owing to the IRAK4-IRAK1-IKKa kinase cascade (59). Liu et al. (60) showed that the mRNA expression levels of TLR2, TLR3, TLR4, TLR7, and TLR8 increased in at least one lymphoid tissue or cell after PRRSV infection in pigs.

RIG-I can recognize relatively short, blunt 5′-triphosphate or 5′-diphosphate double-stranded RNA regions (61, 62), as well as RNA sequences containing complex secondary structures, such as the 5′/3′ UTR containing A/U-enriched group sequences (63, 64). Xie et al. (65) found that the pseudoknot region of the 3′ UTR of PRRSV could act as a pathogen-associated molecular pattern recognized by RIG-I and TLR3 to induce the production of type I IFN. Jin et al. (66) uncovered the relationship between DDX18 and Nsp2 as well as Nsp10; the binding region of DDX18 is found in the N-terminus of Nsp2 and in both the N- and C-terminal regions of Nsp10. When expressed in MARC-145 and primary PAMs, Nsp2 or Nsp10 causes DDX18 to shift from the nucleus to the cytoplasm, augmenting viral reproduction, whereas inhibition of DDX18 gene expression in MARC-145 cells decreases PRRSV replication. Li et al. (67) demonstrated that DDX19A is a new cytoplasmic RNA sensor, which can bind PRRSV RNA and activate the NLRP3 inflammasome to induce the secretion of IL-1β. After pattern recognition receptors detect the conserved sequence of PRRSV, they initiate a series of chain reactions and induce the synthesis and secretion of IFN-stimulating genes (ISGs), type I interferons (IFN-α/β), and various inflammatory cytokines and chemokines (68, 69). Porcine reproductive and respiratory syndrome virus most likely infects cells of the mononuclear/macrophage lineage (70). Miller et al. (71) demonstrated that PRRSV invasion of PAMs observably reduced the ability of these cells to respond to TLR3 stimulation. Preliminary studies have shown that in MARC-145 cells and PAMs infected with the virus, IFN production can also be significantly inhibited; PAMs infected with PRRSV show delayed production and slight activation of type I IFN (72–74). Furthermore, PRRSV replicates and proliferates in the lungs, but IFN-α is almost undetectable in PRRSV-infected pigs (71, 75). This suggests that PRRSV inhibition of IFN production is mediated by RIG-I (76). Spilman et al. (77) demonstrated that PRRSV can induce the formation of a double-membrane vesicle structure, that can transport and conceal viral RNA from detection by host cells, thereby delaying IFN production. Type I IFN expression can be downregulated by PRRSV (78), despite its significance as a crucial component in the antiviral response, preventing the body from effectively clearing the invading antigen, allowing PRRSV to persist in the host for an extended period and evade the host immune system attack, leading to repeated host infections (79). Lin-Lin (80) found that PRRSV infection caused significant immune suppression in pigs in the early stages, with pig immunity gradually showing a trend of recovery after 7–10 days. The PRRSV infection causes respiratory dysfunction in pigs mainly because it induces the expression of several inflammatory cytokines (IFN-γ, IL-10, IFN-α, IL-1β, IL-6, and IL-12), which leads to lung tissue damage. MARC-145 cells infected with PRRSV did not show any change in IFN-α and IFN-β expression, but the cells treated with exogenous double-stranded RNA could produce a large amount of type I IFN. When MARC-145 cells were simultaneously treated with double-stranded RNA and PRRSV, they initially produced type I IFN; however, this response was quickly and strongly inhibited (74). Other studies have shown that PRRSV inhibits the activation of IFNs and suppresses their downstream signaling pathway genes (ISGs), thereby hindering the immune response (81). Zinc finger CCCH-containing antiviral 1 (ZC3HAV1) is an ISG of an antiretroviral factor, also known as zinc finger antiviral protein (ZAP) (82). The main ZAP-mediated antiviral mechanism is to recognize viral RNA and recruit 5′ and 3′ mRNA, using an RNA decay mechanism to degrade the target RNA (83). Following PRRSV infection, ZAP synthesis increases, thereby inhibiting its replication (84). Nsp9 has been reported to interact with ZAP, and PRRSV can evade the restriction imposed by Nsp4 on ZAP, thereby making ZAP reliant on its protease activity. Further studies showed that the degradation of ZAP was dependent on the 180th amino acid (serine) of Nsp4, and that the mutation of residue 180 was very important for the degradation of ZAP (85). Infection with PRRSV can downregulate the expression of type I IFN in the host and facilitate escape from the innate immune response. Many studies have reported that the Nsp1α, NSP1β, Nsp2, Nsp4, Nsp11, and N proteins of PRRSV can play a role in inhibiting innate immune response through different mechanisms (16, 24, 86, 87). These proteins inhibit type I IFN synthesis (88). Nsp1 can inhibit IFN expression by degrading intracellular CREB-binding protein and may also be involved in regulating subgenomic mRNA synthesis (69). Nsp1α can inhibit the phosphorylation of IκBα, thus blocking the nuclear transcription process of NF-κB and inhibiting the activation of IFN-α (89). Nsp1β can inhibit IRF3 nuclear translocation and phosphorylation (15, 90). Nsp2 is a membrane-anchored protein with cysteine protease activity; cysteine protease activity inhibits IRF3 phosphorylation and nuclear translocation and inhibits IFN-β production by blocking IRF3 phosphorylation (91). It also has uncoupling activity that can correlate with ovarian tumor (OTU) domain protease activity. This uncoupling activity is thought to play a role in ISG and NF-κB signaling. NSP4 cleaves the downstream adapter MAVS at Glu268, thereby suppressing type I IFN signaling (92). Wang et al. (93) found that Nsp11 inhibits IFN-I synthesis through a nuclease (NendoU) and inhibits ISRE promoter activity to promote ISG transcription. Nsp11 can inhibit the phosphorylation of IRF3 and IκB, which inhibits the nuclear translocation of IRF3 and NF-κB, thereby limiting IFN synthesis (94). Nuclear factor-κB is a signaling molecule that regulates innate immunity. Blocking NF-κB is a method used by many viruses to evade host innate immunity. In the late stage of PRRSV infection, it can activate the NF-κB pathway in host PAMs and MARC-145 cells, thereby regulating host cell proliferation and apoptosis and optimizing its own replication. However, blocking NF-κB activation by overexpressing the dominant negative form of IκBα did not alter the replication state of the virus. This suggests that PRRSV may be involved in other cellular activities, such as cytokine regulation or apoptosis, when activating the NF-κB pathway (76, 95). Snijder et al. (152) found that Nsp2 and Nsp3 may play a role in the rearrangement of replication-associated membranes. Detailed analysis showed that NSP11 overexpression blocked IFN signaling by affecting the C-terminal-associated domain of IRF9. In contrast, IFITM proteins can inhibit a variety of viral infections through multiple signaling pathways, including restricting viral entry, reducing the expression level and protein synthesis of virus-related genes, inhibiting viral assembly, and reducing viral infectivity (96). Porcine reproductive and respiratory syndrome virus inhibits not only type I IFN signaling but also IFN secretion. When ISG15 and ISG56 mRNA were used as indicators in IFN-α-induced MARC-145 cells, the JAK–STAT pathway was inhibited and ISGF3 nuclear translocation was blocked at 24 h after infection (78). When plasmacytoid DCs (pDCs) were stimulated by TGEV, PRRSV inhibited the nuclear translocation of STAT1 and Nsp1β protein played a dominant role in this inhibition; Nsp1β also inhibited the nuclear translocation of ISGF3 and the phosphorylation of STAT1 (26, 78). However, mechanisms underlying the effects of PRRSV infection on ISGs are not fully understood. In MARC-145 cells, the ability of type I IFN to inhibit PRRSV infection is highly correlated with the expression level of the MxA promoter and less correlated with the induction of IRF3 and IRF7, implying that MxA may be a biomarker for IFN induction during PRRSV infection (97). ISG15 is a ubiquitin-like molecule that reversibly binds to proteins and mediates innate immune responses against viruses. The OTU domain in Nsp2 of PRRSV has deubiquitination activity. Therefore, Nsp2 can reduce the ubiquitination and ISGylation of 293 T cells, indirectly indicating that Nsp2 may help the virus escape the host innate immune response by uncoupling (98). The autocrine and paracrine functions of type I IFN and other related cytokines contribute to immune responses in the body. Some studies have shown the presence of apoptotic cells in PRRSV-infected pig tissues (99); however, in other reports, apoptotic cells were not caused by PRRSV infection (100, 101). Based on various observations, GP5 is reported to induce apoptosis by acting downstream of B-cell lymphoma-2 (Bcl-2), and the apoptotic region is localized to the N-terminal of 119 amino acids of GP5 (102). However, other researchers have proposed that bystander cells undergo apoptosis in response to PRRSV infection, and microarray data in MARC-145 cells indicate only a small increase in pro-apoptotic gene transcription in PRRSV-infected cells compared to that in uninfected cells. However, alternative research suggests that bystander cells may undergo apoptosis in response to PRRSV infection. Nevertheless, the transcription of pro-apoptotic genes exhibits only a marginal increase in PRRSV-infected cells compared to that in uninfected cells, as confirmed by microarray data obtained from MARC-145 cells (103). Subsequent research using monocytes and porcine alveolar macrophage-derived dendritic cells have shown that the stage of viral infection determines whether PRRSV-infected cells are anti- or pro-apoptotic (79, 104). At 8 h post infection, the balance shifted to anti-apoptosis; however, all infected cells eventually died from necrosis or apoptosis. The induction of a strong anti-apoptotic state is inseparable from the expression of viral proteins, and non-structural proteins may be involved in suppressing apoptosis because PRRSV-mediated anti-apoptotic effects occur before the formation of this structure. Studies have shown that IFITM1, a member of the IFITM family, can limit the spread of viruses. Additionally, PRRSV Nsp3 interacts with IFITM1 to induce proteasome-dependent degradation of IFITM1, thus counteracting the restriction of IFITM1 (105). Linear ubiquitin-specific deubiquitinases (OTULIN) control immune signal transduction pathways by restricting Met1-linked ubiquitination. The OTU domain enables porcine OTULIN to interact with NSP11. NSP11 accelerates the linear ubiquitination and degradation of NEMO by recruiting OTULIN, resulting in the additive inhibition of IFN-I production (106). PCSK9 is the ninth member of the kexin-like pro-convertase subtilisin family and plays a role in the maturation of various proteins (107). Wang et al. (108) demonstrated that PCSK9 is synthesized and secreted at the initial stage of PRRSV infection with PAMs, which inhibits PRRSV replication by targeting the viral receptor CD163, and plays an important role in the process of PRRSV infection. Nsp11 antagonizes the antiviral activity of PCSK9, which depends on its endonuclease activity (109). The mRNA capping enzyme 1a (DCP1a) is a cofactor, a protein in the eukaryotic processing body (P-body), involved in the formation, maintenance, and development of P-body (110), and has the function of eliminating the 5′-methyl guanoside cap in eukaryotic mRNA (111). Schoggins et al. (112) and Dougherty et al. (113) showed that DCP1a inhibits translation by activating double-stranded RNA-dependent protein kinase, thereby restraining poliovirus entry, and that DCP1a also inhibits PRRSV, although the mechanism of action has not been elucidated (114). To counter the inhibition of DCP1a, PRRSV utilizes Nsp4 to cleave DCP1a. The cleavage site is glutamate 238 (E238) of porcine DCP1a; it is worth noting that this cleavage site is species-specific. Cholesterol-25-hydroxylase (CH25H), an ISG-encoded polymeric membrane protein, is relatively stable and inhibits the development of viruses through various pathways (115). CH25H restricts PRRSV replication by degrading NSP1α through the ubiquitin-proteasome pathway, with K169 (lysine residue) in the Nsp1 protein being the main ubiquitination site (116). After stimulation by CH25H, the PRRSV E protein degrades CH25H through the ubiquitin-proteasome pathway, and the ubiquitination site was identified at Lys28 (117). NSP11 and Nsp1β exhibit antagonistic antiviral activity against CH25H through lysosomal degradation (118). TRIM25 inhibits PRRSV replication and co-immunoprecipitation experiments have shown that TRIM25 competes with N proteins to interfere with TRIM25-RIG-I interactions. The N protein can decrease the expression of RIG-I mediated by RIM25 and TRIM25, thereby suppressing IFN-β production (119). In summary, PRRSV inhibits natural immunity in the body through various mechanisms.

### 3.4 Antibody-dependent enhancement (ADE) effect of PRRSV

Infection by PRRSV induces an ADE effect, in which PRRSV-specific maternal antibodies or vaccine antibodies not only fail to effectively block viral invasion but also enable viral invasion of target cells and enhance infection (120). Currently, ADE has been confirmed in a variety of viruses such as dengue virus (121) and equine infectious anemia virus (122). Studies have shown that ADE caused by different PRRSV strains also differs. The ADE phenomenon of 17 strains of PRRSV type 1 isolated by Yoon K J (123) was analyzed, and the proliferation of these 17 strains was found to vary. Comparing within and between groups, the viral replication of ISU-P, AZ-1, and KS strains was found to significantly differ in neutralizing and sub-neutralizing antibody titers (*p* < 0.01). The nucleocapsid (GP7), capsid (GP5), and GP3 proteins of PRRSV exhibit strong immunogenicity; however, Christianson et al. (124) conducted an analysis of the relationship between PRRSV epitopes and ADE using monoclonal antibodies and found a positive correlation between the nucleocapsid and capsid proteins and the ADE phenomenon. The synthesis of neutralizing antibodies is mainly induced by GP3. The capsule membrane protein (GP5) could simultaneously induce neutralizing antibodies and was also the primary cause of ADE, vaccine-induced antibodies targeting GP5 of a specific vaccine strain often lack sufficient cross-neutralization activity against heterologous field strains. When such sub-neutralizing antibodies encounter genetically divergent wild-type PRRSV, they bind to viral particles to form antigen–antibody complexes without completely abrogating viral infectivity. Halstead et al. (125) have proposed that various viruses have distinct pathogenic and ADE mechanisms. Currently, the widely recognized mechanism of ADE of PRRSV involves the virus and antibody binding to produce an antigen–antibody complex. The Fc segment of the antibody binds to the cell surface Fc receptors (primarily FcγR, the Fc receptor of IgG) and enters host cells (mainly macrophages of the lung and other tissues) through endocytosis. The virus enters the smooth endoplasmic reticulum by budding with the nucleocapsid. The Golgi apparatus completes the replication and assembly of the virus, which then accumulates in vesicles and migrates to the cell membrane, ultimately being released through membrane fusion. Nagashima et al. (126) found that antibodies can enhance phagocytosis of cells through the mediation of FCγR, resulting in the easy entry of the antigen–antibody complex into cells. Sapinoro et al. (127) studied the enhancement of virus susceptibility to host monocytes and macrophages through cell surface FCγR mediation when antibodies were unable to completely neutralize the virus. Tian et al. (128) found that PRRSV increased its susceptibility to host cells (macrophages) by mediating Fc fragment of IgG, low affinity II receptor (FcγR II). The IL-10 content in bronchoalveolar lavage fluid of PRRSV-infected pigs was significantly increased, possibly because the IL-10 expression of PAMs was upregulated, which led to the decrease in IFN-α/β and other inflammatory factors (129). Halstead et al. (130) reported that the IgG γ chain can stimulate macrophages to produce IL-10 and suppress the secretion of IL-2 and IL-12 through signal transduction, thereby inhibiting the immune response. Furthermore, the anti-inflammatory and immunosuppressive environment caused by the substantial production of IL-10 through autocrine and paracrine mechanisms contributes to viral transmission (131). In summary, the ADE effect of PRRSV can enhance viral infection and cause immunosuppression.

### 3.5 Inhibition of dendritic cell function by PRRSV

Dendritic cells, named for their dendritic shapes resembling tree protrusions, are the most powerful antigen-presenting cells discovered to date and are important contributors during the immune response of the body (132). Dendritic cells have a strong ability to present antigens with an antigen-presenting capacity 100 times greater than that of phagocytes, and can effectively activate initial T cells and serve as an important medium for immunity of the body (133). After being stimulated by the invading foreign antigen, immature DCs in the blood migrate to the infected tissue to acquire antigen information. Through the processing of major histocompatibility complex (MHC) molecules, the phagocytosed antigen becomes an immunogenic peptide and is presented to T cells (134). The DCs participate in the immune response *in vivo* by interacting with T cells. The type of secreted cytokine determines whether the subsequent immune response is Th1 or Th2 (135). Jin-Ling et al. (136) showed that PRRSV infection promotes the expression of the Th2 cytokine IL-10, inhibits the expression of the Th1 cytokine IL-12, and induces a shift in the immune response toward Th2 polarization. The immunosuppressive mechanism of PRRSV on DCs was revealed by examining the imbalance in DC subpopulations and the dysfunction of DCs antigen presentation, which regulates the response of the body to PRRSV by secreting interferons, activating specific inflammatory cytokines, and initiating specific T-cell differentiation. Porcine reproductive and respiratory syndrome virus can impair DC function; invade monocyte-derived DCs; downregulate the expression levels of related molecules such as CD14, CD11b/c, Major Histocompatibility Complex class I (MHC-I), and Major Histocompatibility Complex class II (MHC-II); and reduce the antigen-presenting ability of DCs (104). Leukocyte antigen class I (SLA-1) can help the host initiate an antiviral immune response; however, PRRSV can disrupt the antigen-presenting pathway of SLA-1. Studies have shown that many PRRSV proteins such as Nsp1α, Nsp2TF, Nsp4, and GP3 can down-regulate the expression of SLA-1 on the cell surface (137, 138). Nsp1α can cause proteasomal degradation of the SLA-1 heavy chain. The reduction in nsp2TF-induced SLA-I expression was associated with the last 68 amino acids to the NSP2TF domain. Nsp4 inhibits B2M transcription by binding to the SLA-I promoter, thereby downregulating SLA-I expression on the cell surface. Plasmacytoid DCs, a crucial type of effector cells, express high levels of antiviral type I IFN in the early stages of innate immunity (139). Borghetti et al. (140) and Lunney et al. (141) demonstrated that when pig pDCs were stimulated with PRRSV *in vitro*, pDCs were unable to secrete IFN-α, IFN-γ, IL-6, IL-8, and IL-12. Moreover, when PRRSV infects monocyte-derived DCs and bone marrow-derived DCs, it regulates the synthesis and secretion of various cytokines and cluster of differentiation for DCs molecules related to anti-PRRSV immunosuppression (142). Calzada-Nova et al. (143) conducted human studies and demonstrated that PRRSV can inhibit the type I IFN and pro-inflammatory cytokine response of porcine pDCs; these cells are the primary source of IFN-α and other inflammatory cytokines and respond rapidly to viral infection. Huang et al. (144) demonstrated that *in vitro* cloning of the porcine DC-SIGN (pDC-SIGN) gene enhanced the transmission of PRRSV between target cells, suggesting that pDC-SIGN may serve as an additional receptor for porcine DCs, facilitating the spread of PRRSV between cells. In summary, the interaction between PRRSV and dendritic cells is also associated with immunosuppression.

### 3.6 Destruction of the thymus by PRRSV

The thymus is the most crucial central immune organ in the body where T cells differentiate and mature. Butler et al. (145) studied the damage caused by PRRSV in the thymus of infected piglets and the series of abnormal body reactions caused by PRRSV. They proposed that “After PRRSV infects antigen-presenting cells in the fetal/newborn thymus, the positive selection of CD4^+^ CD8^+^ double-positive thymus cells become abnormal, resulting in a severe lack of T cells.” This induces a series of abnormal immune responses. Immune disorders in piglets infected by PRRSV occur during the critical window period of immune development. Viral infection leads to an imbalance between positive and negative selection of CD4^+^ and CD8^+^ thymocytes in the fetal/newborn thymus, which affects thymocyte development and results in thymus atrophy. Furthermore, the extent of thymus atrophy caused by PRRSV infection is directly related to the virulence of the viral strains. An atrophied thymus leads to deficiency in the T-cell reservoir, rendering it unable to effectively recognize PRRSV. This study suggests that the immunosuppressive effects of PRRSV are associated with thymus atrophy.

### 3.7 Effect of PRRSV on Tregs

Regulatory T cells are a subset of Foxp3^+^, CD4^+^, and CD25^+^ T cells, which are the key cell types that maintain innate and adaptive immunity in the body, and are involved in the generation and maintenance of peripheral immune tolerance (146). Adaptive (or inductive) Tregs are induced and activated by DCs to secrete high levels of TGF-β or IL-10, thereby inhibiting intense inflammatory responses and attenuating host tissue damage (147). Silva-Campa et al. (148) investigated the use of PRRSV to stimulate monocyte-derived DCs *in vitro* and found that stimulation with PRRSV-1 led to an increase in the number of Foxp3^+^, CD4^+^, and CD25^+^ T cells, as well as an elevation in the mRNA expression of Foxp3, demonstrating that PRRSV was able to induce the production of Tregs. However, stimulation of monocyte-derived DCs with PRRSV type 2 did not induce the production of Foxp3^+^, CD4^+^, and CD25^+^ T cells, suggesting that different PRRSV strains have different immunosuppressive effects on the host. Royaee et al. (149) showed that PRRSV type 1 could promote IL-10 secretion and TGF-β gene expression. Díaz et al. (150) demonstrated that PRRSV type 2 did not alter TGF-β production in pigs. However, IL-10 levels increased in the culture supernatants of PBMC obtained from PRRSV-infected pigs. Therefore, immunosuppression caused by PRRSV infection in pigs may be linked to Treg activation.

## 4 Summary and prospects

It has been 39 years since the introduction of PRRSV, with an average annual cost of hundreds of millions of dollars. The search for effective vaccines to resist virus invasion is ongoing to reduce related economic losses. However, the produced vaccines are unsatisfactory for various reasons. In recent years, the effect of common antiviral drugs has become increasingly weaker under the selectivity of the virus, and therapeutic drugs targeting innate immunity in proteins are speculated as being more promising; therefore, new antiviral mechanisms are urgently needed to develop new effective therapeutic drugs (151). Research focusing on viral proteases (Nsp4, Nsp11) and host factors like CH25H may provide new targets for PRRSV treatment. At present, the high frequency of mutations and easy recombination of PRRSV have led to the emergence of variants, and the molecular mechanisms of PRRSV-neutralizing epitopes, neutralizing epitopes of glycosyl shielding, common neutralizing epitopes of heterologous strains, immunosuppression, and immune protection remain unclear, which greatly limits the development of vaccines. The sources of current NADC30 and NADC34 strains are through recombination of vaccine strains, recombination of vaccine and wild strains, and recombination of wild strains. The prevalence of these strains is partly related to the production management of the pig industry. In the context of strong immune pressure, failure to eliminate PRRSV inevitably induces PRRSV variants. Through continuous research, PRRSV is gradually being understood; however, the detailed mechanisms of PRRSV infection and immune evasion are yet to be fully revealed. Therefore, it is essential to study the immune evasion mechanisms of PRRSV, revealing how it evades natural immunity of the host to develop more potent vaccines to manage and eradicate PRRSV. This is particularly important for vaccines that can effectively trigger or boost the innate immune response in hosts.
